# New Technologies to Improve Pain, Anxiety and Depression in Children and Adolescents with Cancer: A Systematic Review

**DOI:** 10.3390/ijerph17103563

**Published:** 2020-05-19

**Authors:** María Mar Lopez-Rodriguez, Alejandro Fernández-Millan, María Dolores Ruiz-Fernández, Iria Dobarrio-Sanz, Isabel María Fernández-Medina

**Affiliations:** Department of Nursing, Physiotherapy and Medicine, University of Almería, 04120 Almería, Spain; mlr295@ual.es (M.M.L.-R.); afmsalud095@gmail.com (A.F.-M.); ids135@ual.es (I.D.-S.); isabel_medina@ual.es (I.M.F.-M.)

**Keywords:** neoplasm, child, adolescent, cancer pain, anxiety, depression, technology, mobile applications, virtual reality

## Abstract

Pain, anxiety, or depression are very prevalent in children and adolescents with cancer, which is a great challenge for health professionals. Several studies pointing out the positive effect of technology on the management of symptoms have been published in recent years. Considering these studies is important in order to reduce the negative impact on the quality of life of this population. This study aimed to analyze the available evidence and to describe the benefits of the new technologies in the treatment of pain, anxiety, and depression in children and adolescents with cancer. A systematic search using six electronic databases was conducted to identify studies using technological interventions with a focus on pain, anxiety, and depression that were published from 2008 to 2018 including oncology patients from 0–18 years old. Out of the 1261 studies that were identified, five studies met the inclusion criteria for this systematic review. Robots were used in two studies, providing amusement and social interventions that showed significant improvements. Virtual reality, a mobile application, and a videogame were used in three studies and obtained beneficial results in pain and anxiety. The studies included in this review suggest that new technologies can be used as an innovative form of non-pharmacological intervention with therapeutic benefits.

## 1. Introduction

Cancer is the second highest disease that causes the most deaths worldwide. Between 2005 and 2015, the incidence of cancer increased by 33%. During 2015, there was a total of approximately 17.5 million cases detected and 8.7 million deaths [1]. However, the incidence of this disease is different in children and adolescents with respect to adults. Ward et al. [2] established that cancer is the second highest cause of infant death after traffic accidents. The incidence of all types of cancer in children aged 0–14 years old was 135.6 per million in people with malignant tumors between 2001–2010 [2]. Leukemia and nervous system cancer (NSC) had the highest rates of child mortality due to cancer: leukemia represented 36.1% of all cases in children between 0–14 years, and NSC represented 20% in children aged 10–14 years. In addition, lymphoma had a 22.5% of case proportion in adolescents aged 15–19 years. Regarding gender, the cancer incidence in boys, compared to rates in girls, was higher during the time period between 2001 and 2010 [3].

Cancer treatment depends on the type and severity of the specific process. Treatments like chemotherapy have shown effects on the central nervous system resulting in the so-called chemobrain, which includes different symptoms reflecting cognitive decline and affecting quality of life [4,5]. However, the treatment of certain symptoms is common. Thus, for the management of oncological pain, opioids are the most effective drugs and the treatment of choice [6]. In general, 60% of patients with advanced cancer experience pain and 20% suffer from it with an intensity ranging from moderate to severe. In addition to this severe pain, the mental and physical suffering is usually present in at least half of these patients [7]. Thus, pain continues to be a prevalent symptom despite the existence of effective treatments [8].

Furthermore, stress is a symptom with a significant prevalence in children and adolescents with cancer [9]. The origin of stress is found in the changes they suffer as soon as they are diagnosed, since their daily routine is altered: children end up missing classes, lose social relationships, suffer physical complications associated with the treatment, uncertainty about the medical process, and fear of death [1]. Therefore, the pain related to the oncological process in childhood may be accompanied by anxiety about medical procedures and hospitalization, local effects of treatment, separation anxiety and psychological stress. In this situation, the presence and support of family are very important, acting as a component of the care of children and adolescents with cancer. Health professionals, in turn, will support families [2].

In relation to depression, the comorbidity of this problem, along with the oncological pathology, directly influences the increase in the burden of symptoms and mortality. This symptom gains importance for adolescents, showing a growth in the rate of depressive disorders related to cancer. Furthermore, depression is usually associated with pain, also pointing out a probable relationship between both variables in the adolescent population [10]. A recent study of childhood leukemia found that young age had a significant negative relationship with health-related quality of life [11]. Nowadays, considering recent studies on pediatric oncology is important in order to innovate and to use interventions that reduce the negative impact of the disease on the quality of life of children and adolescents [2]. In line with this, the combination of psychological interventions with pharmacological treatment has been shown to be beneficial in reducing pain during procedures or techniques required by children [12]. In addition, several studies [13,14] and a review [15] showing t the positive effect of technology on pain management have been published in recent years, providing continuous information and giving support in certain situations [8]. Likewise, digital interventions have been used to monitor symptoms at home, facilitate patient–clinical communication, and as alarm systems to inform healthcare professionals about the evolution and changes of the clinical process [8]. Therefore, the purpose of this systematic review was to analyze the available evidence and to describe the benefits of new technologies such as mobile applications, video games, virtual reality, or robots in treatment of pain, anxiety, and depression in children and adolescents with cancer.

## 2. Methods

### 2.1. Search Strategy

This systematic review was based on the Preferred Reporting Items for Systematic Reviews and Meta-analyses (PRISMA) guidelines [16]. Moreover, this review followed a structured protocol registered with PROSPERO (International prospective register of systematic reviews: CRD42019130313). We conducted systematic literature searches of six electronic databases: Medline via PubMed, Cinahl, Web of Science, PsycInfo, Dialnet, and Psicodoc. Medical Subject Heading (MeSH) and keyword searches were used to find evidence about technological interventions to improve pain, anxiety, and depression in oncology pediatric patients. Our search strategy included “pain”, “robot”, “robotics”, “mobile”, “mobile apps”, “children”, “adolescents”, “virtual reality”, “videogames”, “anxiety”, “depression”, or “artificial intelligence”. The terms related to symptoms, interventions, and population were combined for the search. Other eligible studies were also identified by searching the cited references from obtained published studies.

### 2.2. Exclusion and Inclusion Criteria

Publications were included if they met the following criteria: (1) studies including a pediatric sample (0–18 years old) diagnosed with any type of cancer; (2) published from January 2008 to December 2018; (3) involving outcomes regarding pain, anxiety, or depression in cancer using technological therapies; and (4) published in English or Spanish. Therefore, articles were excluded if they (1) were not a primary research report (e.g., editorials, opinions, case studies, and reviews); (2) were study protocols or measurement development studies; or (3) they did not use pain, anxiety, or depression as variables and did not use technology-based therapies as interventions.

### 2.3. Screening Process

To carry out this review, two authors (M.M.L.-R. and A.F.-M.) independently searched the literature and reviewed all the studies. Data were extracted into standardized forms and verified for accuracy and completeness by the other two authors (M.D.R.-F. and I.D.-S.). Any division of opinions was resolved by consensus. A pre-selection of the documents was carried out by considering if they were within the proposed topic of the study. After eliminating the duplicates, the relevance evaluation was established (verification based on abstracts). After that, a selection of full-text articles was carried out. All articles that did not meet the aforementioned inclusion criteria were excluded. The full text of the studies that met the inclusion criteria was read, analyzed, and included in this review. The reviewed studies implemented a wide variety of interventions and assessment methods and, therefore, meta-analysis was not possible.

One author (A.F.-M.) extracted key data from the included articles in a standardized Microsoft Office Excel sheet. A pilot test of our template was carried out using the first three included articles. Once finalized, a second author (M.M.L.-R.) verified the extracted data using this template. During this verification process, the accuracy and correctness of the extracted data were considered. Upon availability, the following data were extracted from each article: study design, participants and sample size, evaluation method (immediate, previous, or subsequent effects), type of intervention, therapy developed in the control group (if any), the main measurements, and the main results of the experimental group. A non-quantitative synthesis of the extracted data was performed. Finally, in order to solve any incongruence in any study data, the corresponding author was contacted.

### 2.4. Methodological Quality Evaluation

To assess the methodological quality of the included studies, the PEDro (Physiotherapy Evidence Database) scale was chosen [17]. This scale analyzes the methodological quality of clinical trials and has 11 items that can be answered as YES (Y) or NO (N) as well as a total score that varies from 0 to 10, depending on whether the quality is low or excellent. The 11 criteria evaluated in the PEDro scale are: (1) specified eligibility criteria; (2) random assignment; (3) hidden assignment; (4) homogeneity of the groups at the beginning of the study; (5) blinding of subjects; (6) blinding of the therapist; (7) advisor blinding; (8) follow-up of subjects (at least 85%); (9) intent to treat analysis; (10) group statistical analysis; and (11) measures of variability and specific measures. Studies are considered high quality when the scores obtained exceed 5 (6–8: good, 9–10 excellent); moderate quality if the score is between 4 and 5 (fair study); and of poor quality if the score is less than 4 (poor study).

## 3. Results

**Figure 1:**
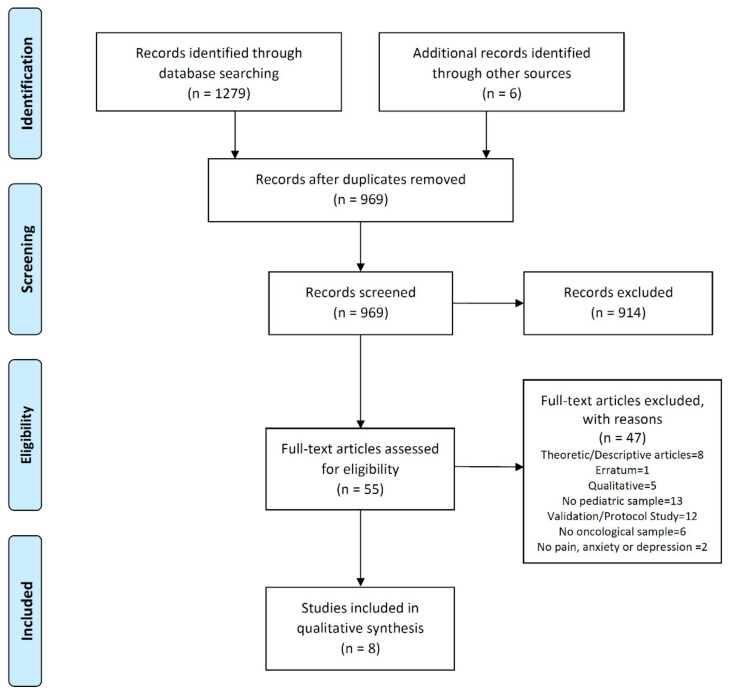
Study flow diagram.

Initially, the search retrieved 1255 references. In addition, we included six additional studies from the bibliographies of articles. After deduplication, the number of references was reduced to 947. After the selection based on title and abstract, 914 articles were excluded. Finally, 33 complete texts were reviewed, which, based on the exclusion criteria of this review, were reduced to eight eligible studies. A flowchart of the study selection process and the reasons for the exclusion are shown in Figure 1.

This review analyzed four randomized clinical trials [18,19,20,21], one no randomized clinical trial [22], two quasi-experimental (non-equivalent control group), one pre–post-test between-subject design [23,24], and an uncontrolled pilot study [25]. In total, the eight studies reviewed had 286 patients. One of the included studies was carried out in an ambulatory setting [25] and the rest in a clinical setting [18,19,20,21,22,23,24]. In addition, two randomized controlled studies conducted follow up [20,25] (Table 1).

### 3.1. Publication Data

All the studies finally included in this review were published between 2009 and 2018. The two studies by Jibb et al. [18,25] were published in the medical journal ‘Pediatric Blood & Cancer’, which has with an impact factor of 2646, is currently located in the first quartile (26/124 in Pediatrics, 33/71, in Hematology, 139/223 in Oncology), and was the journal with the best quality indexes included in this review [26]. Similarly, the study by Atzori et al. [21] was recently published in the journal ‘Frontiers in Psychology’, which is also located in the first quartile, and has an impact factor of 2089 (39/135 in Psychology) [26]. The study by Li et al. [23] was published in the ‘Journal of Clinical Nursing’, which has an impact factor of 1.757 and is located in the first quartile. Another study by Li et al. [24] was published in the ‘Journal for specialists in Pediatric Nursing’, which has an impact factor of 1.130 and is located in the second quartile. The study by Nilsson et al. [22] was published in the ‘European Journal of Oncology Nursing’, which has an impact factor of 1.697and is also located in the second quartile. On the other hand, the study by Fazelniya et al. [20], published in the ‘Iranian Journal of Nursing and Midwifery Research’, lacked this type of quality index. Finally, the study by Alemi et al. [19] that was included in this review was not published in any scientific journal, but in an international conference on robotics that took place in Australia.

### 3.2. Participants and Interventions

The total number of participants among all the analyzed studies was 286. Their ages ranged from four to 18 years, with an average of 9.2 years. These data did not include those of the study by Fazelniya et al. [20], since there was no specified age of the participants. Regarding the gender of the sample, the total percentage of girls was 62.6%. The eight included studies analyzed pediatric patients with active cancer. A single study mixed children with and without cancer [21] (participants without cancer, but with hematological diseases), and none of the studies specified a type of cancer among their selection criteria. Due to an incongruence of the sample size in the study of Alemi et al. [19], the corresponding author for the investigation was contacted.

Regarding the technology used in the different interventions, one study was carried out with a smartphone [25], five studies used multiple devices (virtual reality device with helmet and headset, computer tablet, and computer for video games) [20,21,22,23,24], and two of them included robots as part of the therapy [18,19]. In the studies by Atzori et al. [21] and Nilsson et al. [22], virtual reality was used as a distracting intervention during a venous puncture, comparing it with the standard of care in this type of invasive techniques. The studies by Jibb et al. [18] and Alemi et al. [19] used robotics technology, exposing the participants to interact with the humanoid robots in controlled environments through different sessions [19] and as a distraction during invasive techniques [19]. In the study by Fazelniya et al. [20], the intervention consisted of using videogames with different characteristics (stories with different scenarios and music, puzzles, mission games, etc.). Li et al. [23,24] used videogames in virtual reality with different situations and problems to solve in groups. Finally, Jibb et al. [25] used a smartphone that allowed access to an application specifically designed by researchers to record pain and to give patients guidelines for its management. 

In short, regarding the technology used in the different interventions, one study was carried out with a smartphone [25], five studies used multiple devices (virtual reality device with helmet and headset, computer tablet and computer for video games) [20,21,22,23,24], and two of them included robots as part of the therapy [18,19] (Table 1).

### 3.3. Measures and Results

A total of five studies analyzed pain [18,20,21,22,25], three measured anxiety [19,20,23], and three studies included depression among its study variables [19,23,24]. The variable pain was measured with the Analogue Visual Scale (VAS) in the study by Atzori et al. [21]; Color Analogue Scale (CAS), Facial Affective Scale (FAS), and the Face, Legs, Activity, Cry and Consolability scale (FLACC) in the study by Nilsson et al. [22]; BRIEF PAIN INVENTORY (BPI) in the study by Jibb et al. [25], and with the Faces Scale for Pain-Revised (FPS-R) in the study by Jibb et al. [18]. Regarding the level of anxiety, this was measured with the Multidimensional Anxiety Scale for Children (MASC) in the study by Alemi et al. [19] and with the short form of the Chinese Version of the State Anxiety Scale for Children (CSAS-C) in the study by Li et al. [23]. However, both variables, pain and anxiety, were measured simultaneously with the Pediatric Quality of Life Inventory (PedsQL) scale in the study by Fazelniya et al. [20] given that this instrument includes specific dimensions for pain and anxiety in pediatric cancer in its inventory. On the other hand, depression was addressed using the Depression Inventory for Children (CDI) in the study by Alemi et al. [19] and the Center for Epidemiologic Studies Depression Scale For Children (CES-DC) in the studies by Li et al. [23,24]. Other variables quantified in the studies reviewed were the level of anger, measured with the Anger Inventory for Children (CIA) in the study by Alemi et al. [19]; nausea and fun of patients, measured with VAS in the study by Atzori et al. [21]; quality of life and self-efficacy, measured with PedsQL and General Self-Efficacy Scale (GSE-Sherer), respectively, in the study by Jibb et al. [25]; stress and fear quantified by the Scale of Avoidance and Stress Behavior (BAADS) and the Children’s Fear Scale (CFS), respectively, in the trial by Jibb et al. [18] (Table 1).

A total of three studies [20,21,25] obtained positive results reducing pain levels [20,21], and three [19,20,23] also managed to reduce levels of anxiety and depression [24] after applying their interventions. In one of these studies [21], they found no significant differences in measuring nausea levels. Two studies [18,22] found no significant differences when intervening in pain levels with respect to the control group, and nor did Jibb et al. [18] obtain positive results in the reduction of stress and fear using a robot. However, Jibb et al. [25] obtained improvements in quality of life and self-efficacy, although these were not significant.

### 3.4. Intervention Period and Follow up

The period of intervention of the studies ranged between five and 28 days [19,20,23,24,25]. There was a follow-up period in a single study that lasted four weeks [19]. In a total of six studies [18,19,20,23,24,25], measures were carried out pre-test and post-test. Two studies did not present an intervention period and used a specific intervention [18,21]. The pre-test was carried out before the first intervention session of the study by Alemi et al. [19] and before starting the intervention in the studies by Fazelniya et al. [20] and Jibb et al. [18,25]. The study of Nilsson et al. [22] carried out pre-test, assessment during the intervention, and post-test with a quantitative evaluation and qualitative interview. The study of Atzori et al. [21] did not carry out a pre-test.

The post-test was carried out after three weeks of the intervention period in the study by Alemi et al. [19] and after four weeks in the study by Fazelniya et al. [20], while Jibb et al. [25] conducted a post-test after 28 days of intervention. Atzori et al. [21] applied a single measurement immediately after the end of the intervention. Li et al. [23,24] divided their study in two phases (phase 1 pretest and phase 2 post-test) with a wash out period between them, and Jibb et al. [18] conducted a previous measurement of the level of pain felt by patients in previous venous punctures, and an immediate post-test after the end of the intervention, which consisted in carrying out measurements of three variables in which the patients, their parents, and the nurses participated, who were in charge of carrying out the invasive techniques performed during the intervention period. In this way, three different measurements were obtained within the post-test (Table 1).

### 3.5. Methodological Quality Evaluation

In total, the methodological quality of four randomized clinical trials with a control group and pre-post pilot study of a single group was evaluated. The highest scores on the PEDro scale were obtained by the study of Atzori et al. [21] who obtained six points; Fazelniya et al. [20] with seven points; and finally the study by Jibb et al. [18] that obtained a score of eight, being the highest of all of the articles reviewed in our work. The minimum scores were obtained in the study by Alemi et al. [19] and by Jibb et al. [25]. Thus, the methodology of Jibb et al. [25] was based on the use of a single group with an intervention period that lasted 28 days and an evaluation (post-test) (Table 2).

Among the observed biases, we highlight the absence of a control group in one of the studies [25]. It is also worth mentioning the absence of masking systems in most of the publications [19,20,21,25] as well as the use of very small samples, thus making their results not generalizable. Likewise, it is important to highlight the short period of follow-up in the study by Fazelniya et al. [20] and the fact that the study participants in Alemi et al. [19] knew the members of the research team before the trial began.

## 4. Discussion

### 4.1. Main Findings

The aim of this review was to carry out an evaluation of the effectiveness of new technologies in the treatment of pain, anxiety, and depression in children and adolescents with cancer. These technologies consisted of the development of applications for smartphones, videogames for tablets or computers, virtual reality equipment, and robots specifically designed to perform interventions in the field of pediatric oncology. The results of four randomized clinical trials, one non randomized clinical trial, two quasi-experimental studies, and a pilot study without a control group were included in this review [18,19,20,21,22,23,24,25]. Due to the scarcity of articles on robotics in this topic, one of the studies reviewed [19] was not published in a scientific journal, but published at an international congress. Most of the original studies demonstrated the beneficial effects of new technologies on pain, depression, or anxiety as well as other secondary variables that were included (nausea, fear, stress, anger, self-efficacy, and quality of life). The intensity of pain was significantly lower in patients exposed to videogame or virtual reality interventions [20,21]. However, one of the analyzed studies did not find significant differences [22]. Of the two studies that used robotics, one of them found significant results [18] in the improvement of pain using robots as a positive distraction during an invasive technique. Anxiety and depression were used as variables in the other study on robotics [19] and virtual reality computer games [23,24], which found significant results regarding improvement when applying the intervention. Li et al. [23] did not obtain significant results in anxiety reduction. Likewise, both the pain and quality of life improved with the use of a mobile application [25].

The structure of the application consisted of online questionnaires to assess pain intensity, management, and interference with needs that may affect quality of life (such as sleep). Through the application, patients received real-time self-care recommendations and a subsequent reevaluation to verify its effectiveness. Although Jibb et al. [18] demonstrated the feasibility of using distractor robots during invasive techniques, these authors did not obtain significant results in the reduction of pain levels, but did obtain benefits in stress levels. Nevertheless, the authors of this study related these results to the small sample size and thought that it would achieve a change in pain intensity in a larger study.

### 4.2. Comparison with Existing Literature

To the best of our knowledge, this is the first systematic review that deals specifically with the use of new technologies (including mobile phones, robotics, video games, or virtual reality) to improve pain, anxiety, or depression in pediatric patients and adolescents with cancer.

Although some systematic reviews that deal with the topic of oncological pain and its management with applications of smartphone or with technologies in general have already been published, there are few publications related to robotics in relation to health, especially in a specific way in the pediatric population. Robotics is more widespread in its use within medical-surgical specialties, so during our literature search, most of the publications on robots that appeared in the databases were based on robotic innovations applied to certain types of operations in the operating room. In this way, robots used within the field of pediatric oncology to obtain improvements in symptoms such as pain, anxiety, and depression are not a frequent intervention tool. In general, most studies in relation to new technologies in health have focused on technological interventions as a method of distraction during invasive techniques such as robotics during vaccinations or venous punctures [18,22,27]. Regarding videogames, we did not find any systematic review that focused on its effect on symptoms in children and adolescents with cancer. Govender et al. [28] revised the therapeutic use of videogames, virtual reality, and mobile technology and studied these technologies on pediatric patients with cancer in a general way, developing a narrative review of the different tools that are available to use on children with this pathology without specifying variables. In another review [29], videogames were studied as therapy in the promotion of physical activity to improve variables related to health. This publication did not use an oncological sample and the participants were not exclusively children or adolescents [29].

Virtual reality has been studied more frequently [12,30], and there is a literary review of its effectiveness as a distraction to reduce pain and anxiety in children [31]. The authors of this review worked on both acute and chronic pain in pediatric populations and conceptualized virtual reality as a novel technology that offers the opportunity to modulate the experience of pain in a unique way. The main difference with our work lies in the fact that Won et al. [31] did not develop a systematic review and did not focus on patients with cancer. In addition, we worked with different types of technology, thus being more specific. The systematic review published by Hernández et al. [15] was oriented toward the use of mobile applications to improve pain or fatigue in people who had overcome some type of cancer, and showed the mainly beneficial findings of the different studies they reviewed in their results. In parallel, our review also used this type of technology, but there were important differences in the type of population, since Hernández et al. [15] did not exclusively use a pediatric sample in addition to their participants having already overcome the disease.

Cultural aspects and high cost could also be an obstacle in the use of these technologies for pediatric cancer patients. On one hand, we observed that the included studies did not address cultural aspects capable of influencing receptivity to the different technologies. Only in Alemi et al. [19] were certain difficulties in recruiting participants specified due to the novelty of the intervention and the scarcity of psychological therapies in Iranian hospitals. On the other hand, regarding cost-effectiveness, the analyzed studies did not address the cost of the technological interventions they applied. However, in the study by Jibb et al. [25], the possibility of carrying out a cost-benefit analysis on its mobile application in a future project was mentioned. Along this line, a recent review [32] concluded that the investment in this type of tool development is very high. Furthermore, the real availability of these instruments considered in the study’s methodology was pointed out in this review [32]. Thus, more studies addressing cost-effectiveness of new technologies in general and robots are necessary, in particular, applied to pediatric oncology.

The preferences of users and their receptivity regarding the types of applications they download were addressed in the study by Do et al. [33]. The authors concluded that young subjects show less interest in health-related applications; on the contrary, the most widely used apps were those related to beauty advice and disease prevention. Furthermore, quality of life stood out as a possible influence with a greater tendency to use health-related applications in participants with a lower quality of life. In addition to the thematic, the type of presentation of the applications could influence the receptivity in young patients and their effectiveness as an intervention [33]. Therefore, the importance of the design and aesthetics of the applications has been pointed out as a factor that should be considered with greater emphasis. Finally, the participation of health professionals would be relevant to develop this type of technology in healthcare [34,35]. In line with Zhang et al. [36], for pediatric cancer patients, these types of applications should combine music with visual elements, fusing symptom assessment with direct interventions that promote self-care and reduce stress levels [25,33].

Although cognitive deficits (such as those in the chemobrain) along with physical or psychological changes have been associated to the type of treatment, only one of the studies in this review, that by Fazelniya et al. [20], controlled the effect of this variable in their results. Therefore, the rest of the studies included patients with different type of treatments in their samples. This heterogeneity in the sample could be a limitation to understanding the effect of each intervention on different variables. In addition to type of treatment, gender, age, and even ethnicity could influence the evolution of the patient. Along this line, girls have shown more anxiety and depression than boys; adolescents experience the disease worse because of social changes, and the different treatments affect quality of life depending on the side effects they produce [37,38]. However, in general, these mediating variables were not considering in the analyzed studies. Nevertheless, Atzori et al. [21] pointed out fun as a mediating variable and stated no gender differences in their results. In the same way, Fazelniya et al. [20] did not find gender, educational, or disease duration mediating in their results. Finally, Jibb et al. [25] indicated the self-efficacy variable as a possible mediating variable.

## 5. Limitations and Strengths

The main limitations of our study are the scarcity of designs with high methodological quality, the absence of a meta-analysis, and samples of adequate size that provide generalizable results. A large number of studies that have introduced innovative technological interventions (design of humanoid robots, mobile applications with child interfaces, and focused on childhood within a context of oncological disease) are very recent and most are pilot studies of combined methodology, qualitative, or excessively reduced samples that did not produce relevant results. These publications were excluded despite their innovative nature, since most of them aimed to expose the technological development processes of their instruments rather than using them as an intervention in a group of patients to obtain results in relation to our variables of interest. Another limitation is the use of the PEDro scale in a pilot study of pre-post design with a single group. The main strength of our review is that it is based on the PRISMA guide for systematic reviews, having reviewed very recent articles in a hardly explored sample given its specificity, endowing this review with an innovative character.

## 6. Implications and Conclusions

Based on the results obtained, new technologies have the ability to provide an innovative way to treat pain, anxiety, and depression as the main symptoms of childhood cancer. In addition to pharmacological therapy, the development of mobile applications, robotics, or video games and virtual reality can be beneficial as alternative therapies in the handling of these kinds of problems.

In the studies reviewed, beneficial results were obtained through social interaction with robots and its use as a distraction, symptom registration through applications, and the use of video games and distractions through virtual reality. While it is true that more studies are required with better designs that have higher methodological quality and provide us with more significant results, we can state that this is an expanding field of research, where most of the publications are pilot studies or protocols of study and the exhibition of technological development processes. Nonetheless, most studies agree on the following: it is necessary to expand the research and carry out more elaborate scientific designs with representative samples that test robots or mobile applications under controlled conditions, given that the small results of those already available have been positive.

Some of the implications of this study include assessing symptoms reflecting cognitive decline as a consequence of chemobrain in future research [4,5] and considering the moderating variables. Moreover, implications for nursing practice include the use of emerging technologies as new working methods in oncology nursing, especially in the care of pediatric patients. Thus, the transfer of basic activities of nursing care such as pain management toward a more integrating axis addressing the patient’s holistic perspective could occur in the future.

## Figures and Tables

**Table 1:** Characteristics of the sample, protocol, and intervention.

Study	Design	Population	Outcomes	Intervention	Timing	Results	Limitations	Selection Criteria
Alemi M et al. [19]	RCT	11 participants with any type of cancer. 7–12 years (Mean = 9.45) GC: n = 5 GI: n = 10; 4 dropouts Does not specify gender	Anxiety: MASC. Depression: CDI. Anger: CIA.	Use of the Robot NAO in 8 scenarios (8 sessions during 3 weeks) Different roles in each session.	Pretest: Before 1st session Post-test: after 3 weeks of intervention	GI: Decrease anxiety, depression and anger. GC: No differences	Small sample Difficulty to complete 8 sessions They knew the research team before study	Pediatric sample. Participants in active oncological treatment.
Fazelniya Z et al. [20]	RCT with two step test plan of before and after the intervention	64 participants with cancer in active Chemotherapy treatment 8–12 years (Mean = 10.05) GC: n = 32; (Mean age: 10.2) GI: n = 32; (Mean age: 9.9) 30 girls (46.88%)	Quality of Life: PedsQL with specific dimensions for pediatric cancer (pain and anxiety)	GI: Play a Videogame 3 h peer week 1 month GC: Care routine	Pretest: Before intervention Post-test: Immediately after the intervention Follow-up: 4 weeks in the intervention’s period	GI: Improve quality of life after intervention and 1 month of follow-up GC: No changes	Small sample Short follow-up. Use of other ways to education during the study	Pediatric sample with any type of cancer. Ability to read and use videogames. No physical or mental deficits before the disease.
Jibb L et al. [25]	A pilot one-group pre-post study	N = 40 participants with cancer 12–18 years old (Mean: 14.2) 17 girls (43%)	Effectiveness of the mobile App: AES. Pain: BPI. Interference of pain: PROMIS-PPI-SFS Quality of Life: PedsQL. Self-efficacy: GSE-Sherer.	Use an app during 28 days and participants’ valuation after intervention.	Pretest: Before intervention Post-test after Intervention Follow up: 28 days in the intervention’s period.	GI: Improves pain intensity and interference. Good adherence although it was reduced over time	Pilot Study. Small sample. No control group.	Pediatric sample Active oncological treatment at least 2 months Pain registers higher than 3 (VAS) at least once a week prior selection. Not comorbidities. Not final phase of life.
Atzori B et al. [21]	RCT	N= 15 participants with cancer (n = 11) and hematological diseases. (n = 4). GC: n = 15 GI: n = 15 (They all go through both conditions) 5 girls (33.3%) Mean age: 10.92	Pain: VAS Quality of VR: Self-administered questionnaire. Sickness: VAS Fun: VAS	GI: Use of VR during a venous puncture. GC: A conversation as a distraction	A single measurement after the application of the invasive technique.	GI: Improve pain after use VR during invasive technique. Higher level of fun. GC: Higher level of pain. No sickness differences between groups.	Small sample. Use of a care standard as an intervention in the GC instead of a Conventional intervention.	Pediatric sample with oncological and hematological diseases. Participants without physical or mental deficits before disease. Able to wear a helmet and willing to interact with the VR environment.
Nilsson et al. [22]	RCT	42 participants with any type of cancer. 5–18 years (Mean age: 11) GC: n = 21 (Mean age: 11) GI: n = 21 (Mean age: 11) 4 dropouts. 17 girls (40.5%)	Pain: CAS, FAS, FLACC, Heart rate.	VR as a distraction during a puncture.	Test before intervention, during intervention and immediately after the intervention. No follow up.	No significant differences between GC and GI.	Small sample. Difficulty to adapt the VR to the procedure.	Pediatric sample with any type of cancer. No cognitive impairments.
Li et al. [23]	Quasi experimental control group pre-post between-subject design.	122 participants with any type of cancer 8–16 years (No mean age in total group) GC: n = 70 (Mean age: 12.1) GI: n= 52 (Mean age: 11.6) No dropouts. 57 girls (46.7%)	State Anxiety: CSAS-C; Depressive symptom: CES-DC	Game of Virtual reality in groups	Two phases: pre-test (phase 1), a washing period, (1 month), post-test (phase 2). No follow up.	GI: Decrease depressive symptom. No significative differences in anxiety. GC: No changes.	Little generalizable results. Difficulty to participate in intervention after chemotherapy	Pediatric sample with any type of cancer. No cognitive or learning problems.
Li et al. [24]	Quasi experimental control group pre-post between-subject design.	122 participants with any type of cancer. 8–16 years. GC: n = 70 (Mean age: 12.1) GI: n= 52 (Mean age: 11.6). No dropouts. 57 girls (46.7%).	Depressive symptom: CES-DC.	Game of Virtual reality in groups	Two phases: pre-test (phase 1), a washing period, (1 month), post-test (phase 2). No follow up	GI: Decrease depressive symptom. GC: No changes.	Little generalizable results. Difficulty to participate in intervention after chemotherapy	Pediatric sample with any type of cancer. No cognitive or learning problems.
Jibb L et al. [18]	Parallel Clinical randomized trial	N= 40 participants with cancer 4–9 years old. Mean of age: 6.2 16 girls (40%) GI: n = 19 GC: n = 21	Pain: FPS-R Stress: BAADS Fear: CFS	Robot that uses cognitive-behavioral interventions or dances and sings during puncture.	Pretest: Pain level in previous punctures. Post-test: Immediately after the intervention.	There were not differences in pain, fear and stress levels in both groups.	Results difficult to generalize (single site study). Limited robot ability to assess impact on pain	Pediatric sample. In active oncological treatment at least 1 month since diagnosed. No previous physical or mental problems. Previously exposed to venous punctures.

Abbreviations: AES: Acceptability E-Scale; App: Application; BAADS: Behavioral Approach-Avoidance and Distress Scale; BPI: Brief Pain Inventory; CAS: Color Analogue Scale; CDI: Children’s Depression Inventory; CES-DC: Center for Epidemiologic Studies Depression Scale For Children; CFS: Children’s Fear Scale; CIA: Children’s Inventory of Anger; CSAS-C: Chinese Version of the State Anxiety Scale for Children; FAS: Facial Affective Scale; FLACC: the Face, Legs, Activity, Cry and Consolability Scale; FPS-R: Faces Pain Scale-Revised; GSE-Sherer: General Self-Efficacy Scale; MASC: Multidimensional Anxiety Scale for Children; N= Total number of participants; n= Number of participants in a group; PedsQL: Pediatric Quality of Life Inventory; PROMIS-PPI-SFS: Patient Reported Outcomes Measurement Information System Pediatric Pain Interference Short form Scale; RCT: Randomized Controlled Trial; VAS: Visual Analogue Scale; VR: Virtual Reality. Countries where the studies were conducted: Canada [18,25], Iran [19,20], China [23,24], Sweden [22], and Italy [21].

**Table 2:** Methodological quality of the studies according to the PEDro Scale.

Study	1	2	3	4	5	6	7	8	9	10	11	Total
Alemi M et al. [19]	Y	N	N	Y	N	N	N	N	N	Y	Y	4 (fair)
Fazelniya Z et al. [20]	Y	Y	N	Y	N	N	N	Y	Y	Y	Y	7 (good)
Jibb L et al. [25]	Y	N	N	Y	N	N	N	N	N	N	Y	3 (poor)
Atzori B et al. [21]	Y	Y	N	Y	N	N	N	Y	Y	N	Y	6 (good)
Jibb L et al. [18]	Y	Y	N	Y	N	N	Y	Y	Y	Y	Y	8 (good)
Nilsson et al. [22]	Y	N	N	Y	N	N	N	Y	N	Y	Y	5 (fair)
Li et al. [23]	Y	N	N	Y	N	Y	Y	Y	N	Y	Y	7 (good)
Li et al. [24]	Y	N	N	Y	N	Y	Y	Y	N	Y	Y	7 (good)

Abbreviations: 1: The election criteria were specified; 2: The subjects were randomly assigned to the groups (in a cross-over study, the subjects were randomly distributed as they received the treatments); 3: The assignment was hidden; 4: The groups were similar at the beginning in relation to the most important prognostic indicator; 5: All subjects were blinded; 6: All therapists who administered the therapy were blinded; 7: All evaluators who measured at least one key result were blinded; 8: The measurements of at least one of the key results were obtained from more than 85% of the subjects initially assigned to the groups; 9: Results were presented for all subjects who received treatment or were assigned to the control group, or when this could not be done, the data for at least one key result were analyzed by “intention to treat”; 10: The results of statistical comparisons between groups were informed for at least one key result; 11: The study provides punctual and variability measures for at least one key outcome; Score: Each criterion met (except the first); N: Did not meet criteria; Y: Met criteria.
